# How to become a medical professor – a comparative analysis of academic requirements in Germany and the United States

**DOI:** 10.1515/iss-2019-0011

**Published:** 2019-08-22

**Authors:** Seyed Arash Alawi, Rosalia Luketina, Nicco Krezdorn, Lukas Fabian Busch, Anne Limbourg, Ludwik Branski, Peter M. Vogt, Andreas Jokuszies

**Affiliations:** Department of Plastic, Aesthetic, Hand and Reconstructive Surgery, Hannover Medical School, Carl-Neuberg-Strasse 1, 30625 Hannover, Germany, Phone: +49 511 532-8864; Department of Plastic, Aesthetic, Hand and Reconstructive Surgery, Hannover Medical School, Hannover, Germany; Department of Plastic Surgery, University of Texas Medical Branch, Galveston, TX, USA

**Keywords:** academic career, academic system, professorship, research and teaching

## Abstract

**Background:**

The acquisition of a medical professorship represents a significant step in a physician’s academic career. The responsibility as well as the honor and the associated obligations are significant; however, the requirements to become a medical professor vary in Germany.

**Objective:**

We analyzed the variable requirements for prospective medical professors in Germany, with special focus on the tenure track concept and the U.S. system.

**Methods:**

Based on an online research, we queried German medical faculty regulations to obtain a medical professorship within Germany.

**Results:**

We analyzed 35 German universities. On average, 11 publications are required after “venia legendi” to meet professorship (apl) prerequisites (median x̅ = 10, max = 24, min = 6, n = 16), whereas 6 publications with first or last authorship are required on average (x̅ = 6, max = 16, min = 4, n = 26). In most German universities, it takes an average of 4 years after gaining habilitation to apply for a professorship (x̅ = 5 years, max = 6 years, min = 2 years). Candidates for university chair positions, however, can shorten this period by an average of 38%.

**Discussion:**

In the German academic system, the prerequisites to gain a professorship differ among universities. Due to different scientific cooperation and exchange programs, research and academic activities have reached an intense international exchange level. Yet there is no international or even national standardization, quality assurance, and comparability to gain a medical professorship.

## Introduction

### German academic system

If you aspire to become a medical professor in Germany, the stairway is steep; however, there are different ways of climbing up the academic career ladder. As soon as you reach “venia legendi”, specific faculty requirements are set to obtain professorship. For example, among all members of German plastic surgery departments, about 14% of the members of the German Society of Plastic, Reconstructive and Aesthetic Surgeons ( DGPRÄC, Deutsche Gesellschaft der Plastischen, Rekonstruktiven und Ästhetischen Chirurgen) complete their habilitation, and about 7% reach an associate professorship/full professorship [1]. Candidates who are willing to obtain a professorship show special research enthusiasm, persistence, creativity, ambition, and resilience for extended periods. The possible reasons to go the extra mile of an academic career might be curiosity and interest in optimizing treatment modalities. Salary increase as a further motivation has to be considered as second rate, but working in an academic environment with scientific networks may count as an incentive. Fact is that research takes a central role in clinical routine, especially for those who have the ambition to be a senior academic. Further factors of motivation can be the prestige and influence that impact on one’s decision making at the university level. Especially, in university departments, potential political influence and committee membership is limited to esteemed professors. The thrill and burden of high responsibility accompanied usually by massive workload and limited private life are two sides of the same medal.

The management of universities has not only changed drastically in recent years. University and faculty leaders carry an ascending influence as experts in health politics and economics. For example, ministries negotiate target agreements with universities to allocate their funds according to key performance indicators [2], [3], [4]. Fifteen federal states have introduced university councils called “Hochschulräte” as the new governing body [3]. The university’s senate nominates these members. The state ministries of education decide on the recruitment of professors and can reject the submitted candidates. Although the state laws differ considerably, the university council executes three basic functions in varying degrees. It is intended to address the concerns of society, to take over the supervisory functions formerly assigned from the state to the presidium/rectorate, and to advise the university in its strategic development based on the expertise and experience. With this functional spectrum, the university council receives one central role for the development and sustainable management of the respective university [2], [3].

In comparison to the German university system, individual universities in the U.S. higher education system have maximum autonomy in an output-oriented and competitive environment [5], [6]. This reflects the ongoing political discourse of the Federal Republic of Germany regarding the future strategic orientation of universities being forced by the American model, which appears to “strengthen university autonomy” and “introduce a board as a steering committee” [2], [5].

For example, from the perspective of the field of plastic surgery worldwide, we have gone through exciting and innovative developments, especially in the last decades. Plastic surgery departments of high output in Germany have developed a high level of specialization with an academic background that is defined in an independent academic environment within the university [7], [8], [9]. This independence is the base for academic careers. Additionally, funding and financial resources seem to be more available at university hospitals. Current developments include the establishment of a registry of research funding at the DGPRÄC [10]. Currently, significant discrepancies exist between dependent and independent plastic surgery university hospitals regarding material and human resources. Investigations showed that the scientific performances of university hospitals are significantly better [8]. Regarding career steps, most of the habilitated physicians from German universities leave university hospitals before gaining a professorship [1]. The underlying facts in quitting the research field varies and may range from losing interest in research to “burn out” considered as not being able to exhibit the expected results. In addition, reorientation and the desire to work in a private practice count as further reasons [11], [12]. At the same time, the absence of financial and structural incentives leads to a lack of willingness to choose the stony path of obtaining a professorship. Analyses of German plastic surgery departments show that the intention to proceed with academic career stagnates after the accomplishment of habilitation [4].

Regarding the comparable academic positions, a so-called “Privatdozent” in Germany is not completely comparable to an “assistant professor” in the United States, as an assistant professor might not have completed as much research as a German “Privatdozent”. Comparatively, a senior physician in the United States can be termed as an assistant professor (Table 1). A detailed comparison between the U.S. system and the German system seems to be very difficult, yet Table 1 gives a rough comparison of the positions. In addition to the well-known forms of “außerplanmäßiger Professor” (apl professorship) and W2/W3 professorships in the German system, junior professorship qualification also has been established for the purpose of becoming a full professor in Germany since 2002. Junior professors perform the same tasks as regular professors; responsibilities include tutoring and supervising students, running third-party funded projects, and performing committee work and administrative tasks. However, teaching load is reduced compared to full university professors (W2/W3). This provides more time to develop a research profile. The entire term of a junior professorship is usually 6 years. However, junior professors are initially hired for a limited period. The continuation of junior professorships is decided in the context of interim evaluation [13]. In addition, junior professorships are connected with a tenure option based on the U.S. academic system. The tenure track program for the promotion of young scientists should help to make the career paths in the academic world more transparent and to attract more university teacher careers in medicine. If successful, these positions should lead to a regular professorship without being publicly advertised again [14]. This also eliminates the time-consuming appointment procedure for a full professorship. Junior professorship was launched in 2002 with the fifth amendment of the Higher Education Framework Act. The objective of this amendment was initiated by Edelgard Bulmahn, the former federal minister of research, with the aim of making the German science system more competitive, especially at an international level [15]. Due to the continuing brain drain, the best minds were hired away of the German research and innovation location by other countries [16].

**Table 1:** Overview of the German and U.S. academic systems.

(1) German system	(2) U.S. system
Professor emeritus (Prof. em.)	
Professor ordinarius (ordentlicher Professor, o. Prof., Univ. Prof.)	Full professor
Professor extraordinarius (“extraordinary professor”, außerordentlicher Professor, ao. Prof., apl Professor)	Associate professor
Privatdozent (Priv.-Doz. or PD)	Assistant professor (not entirely comparable)
Dr. med.	Research associate, lecturer, and instructor

(1) German system. Professor emeritus is used both for the university ordinarius and for the extraordinarius. Nevertheless, strictly speaking, only the professor ordinarius can be described as such. In spite of retirement, there is the possibility to proceed working in private practice. Extending his or her former ordinariate beyond the regular time frame is a matter of negotiation. Professor ordinarius: If one person holds a university chair, he is called University Professor. Professor extraordinarius: An extraordinary professor is comparable to an associate professor and does not hold a university chair. The title is given to a former Privatdozent who did excellent research before and after habilitation but has not attained a regular chair. The nomination “außerplanmäßig” means extraordinary. Nonetheless, his obligations are mainly teaching and conducting research besides clinical care according to her or his status being a member of the faculty. Privatdozent: The Privatdozent is also member of the faculty and is not completely comparable to an “assistant professor” in the United States, as an assistant professor might not have completed as much research as a German “Privatdozent”. The obligations include teaching, research, and clinical care. A Privatdozent is also allowed to supervise doctoral theses. (2) U.S. system: professor, associate professor, assistant professor, research associate, lecturer, and instructor, adjunct professor/lecturer/instructor.

The aim of the reform was to reorganize the academic career paths in Germany from scratch and to grant young researchers the scientific freedom that would otherwise take them abroad in other countries. This was based on the American science system, which allows scientists to research and teach independently rather quickly after completing their doctorate. One of the core elements of the reform was junior professorship. It was created based on the assistant professors in the American system, with the aim to equip them with a tenure option and transfer them to a regular professorship if the interim evaluation was successful. Simultaneously, the “additional scientific achievements” formulated in the university laws should no longer be part of the examination procedure. This would have meant the end of habilitation. In Germany, teaching is one of the integral constituents of a “Privatdozent” after habilitation, who is further authorized to supervise doctoral thesis. One can apply for the position of apl professorship equivalent to the associate professor in the United States after continuation of research and teaching [17].

University hospitals and hospitals with university association have, besides clinical patient care and research, also the task of teaching. Teaching is an important part of academic work but often takes place alongside patient care and research activity. High-quality and sustainable knowledge transfer with the aim of promoting creative thinking processes and problem-oriented learning should be the aim of modern teaching concepts at university hospitals. The imparting of the ability to critically evaluate and solve medical questions continues to be a challenge for modern teaching institutions. Teaching activities are already graded at various universities. However, these have no relevance to the attainment of habilitation or for the further career steps in most universities. Nevertheless, teaching is required to obtain the various academic degrees and assessed based on hours completed.

After reaching a full professorship, the pay scale “W” regulates the salaries for university professors in Germany and includes grades W1 to W3. The “Professorenbesoldungsreformgesetz” was introduced in 2002 as a substitute for salary class C. The letter “W” stands for science (“Wissenschaft”). The basic salaries in the W salary can be increased by allowances (performance allowances) for W2 and W3. The allowances can usually reach a maximum of 40% of the basic salary [18].

### U.S. academic system

The medical academic system of the United States includes also different kinds of graduations of a professorship. First, a postdoctoral candidate is offered an “assistant professorship”, which is vaguely equivalent to habilitation. In terms of content, the assistant professor does not completely equate to the “Privatdozent”, as every new salaried senior physician automatically becomes an assistant professor in the United States regardless of scientific work or publications. The next step to become an associate professor is much more difficult, with requirements in research, teaching, and clinical work that varies significantly from university to university.

After a probationary period of usually 7 years, the assistant professor is able to receive an associate professorship, which is comparable to an extraordinary professorship (German: apl Professur). In addition, one can earn a “full professorship” position or a “distinguished professor’s” position. These are often coupled with a donated chair, so that alumni, companies, and other foundations provide funding. This kind of medical professorship is more untypical.

To distinguish between the main working focus, a prefix characterizes the position like “clinical assistant professor”, “clinical associate professor”, or “clinical professor”. There are further differences in the nature of the activities. Lecturers can be full-time employees who, however, have no obligation to perform research and publish scientific papers. They are expected to mainly teach. In other words, the “clinical instructor” is the only position involved in research without teaching requirement. Thus, good teachers are not involved with additional research. The promotion from “assistant professor” to “associate professor” is not defined very precisely based on the online information of the universities. Stanford University, for example, describes a qualification for this position as very variable but still demands “the requirement of excellence, however measured”. The requirement of top performance is described without any measurable conditions. Some recurring criteria are regional celebrity. In the case of Stanford School of Medicine, regional is defined as an area of approximately 322 km. When, however, the reputation as an expert is required, it is considered an unspecific soft criterion.

An exact comparison of the systems is difficult and not intended, yet the main differences between doctors working in the United States are often multiple affiliations divided in research, teaching, and patient care. In contrast, physicians in Germany generally only have one contract, in which it is often not explicitly defined to what extent they are active in the respective fields of activity. A translation of the positions in both systems is therefore problematic but should be listed roughly in Table 1.

The conditions for the academic career with habilitation and obtaining professorship are equivalent for all medical subjects of a faculty.

As the requirements to become a medical professor vary in Germany, we aimed to analyze variable requirements for prospective medical professors in Germany. The listed data apply to all medical disciplines of the respective faculties.

Based on an online research, we queried German medical faculty regulations to obtain a medical apl professorship within Germany. We analyzed the variable requirements for prospective medical professors in Germany.

## Materials and methods

We carried out a web-based analysis of available online information about conditions of obtaining an apl professorship based on the regulations of German medical faculties independent of the medical discipline. To work out the differences, we evaluated both faculty regulations and the federal state law and analyzed all updates concerning the subject.

We evaluated (1) the total publications needed, (2) the proportion of first authorship/last authorship, and (3) the time span in years needed to become a apl professor after obtaining “venia legendi” as well as (4) the percentage of time reduction possible for obtaining a full professorship.

A descriptive statistical evaluation was performed using Microsoft Excel (version 2016, Microsoft Office, Microsoft Corporation, Redmond, WA, USA). The data were evaluated under the assumption of being publically available information. An approval by the local Ethics Committee was not necessary.

## Results

Within Germany, the regulations for obtaining a medical apl professorship are updated independently by each federal state. These laws are updated at regular intervals being adjusted by the universities, which in turn update their faculty regulations. The regulations show how the state laws are practically handled and what requirements are needed to apply for an apl professorship or full professorship. Eighteen of 35 German universities mentioned a date for change of the respective law on higher education. The average period of faculty regulation updates, however, exceeded the time point of the state law update by more than 7 years (max=21 years, min=0 months). Table 2 shows the detailed conditions, which are accessible online.

**Table 2:** Overview of the detailed conditions in German universities.

No.	University	Years after venia legendi	Possible reduction	Possible percentage of reduction (%)	Total publications	First author/last author
1	RWTH Aachen University	5	–	–	12	8
2	Charité-University Medicine Berlin	–	–	–	–	5
3	Ruhr-Universität Bochum	5	4	20	10	8
4	University Bonn	5	3	40	12	8
5	Universität Dresden	4	–	–	–	6
6	Universität Duisburg-Essen	5	0	–	10	6
7	Universität Düsseldorf	5	3	40	–	–
8	Universität Erlangen-Nürnberg	6	4	33	–	6
9	Universität Frankfurt	6	–	–	24	16
10	Universität Freiburg	2	–	–	–	–
11	Universität Gießen	–	–	–	–	–
12	Universität Göttingen	3	2	33	8	5
13	Universität Greifswald	5	–	–	–	5
14	Universität Halle-Wittenberg	4	–	–	8	4
15	Universität Hamburg	–	–	–	–	–
16	Medizinische Hochschule Hannover	4	2	50	8	–
17	Universität Heidelberg	–	–	–	–	–
18	Universität Heidelberg/Mannheim	2	–	–	–	4
19	Universität Jena	5	–	–	10	5
20	Universität Kiel	4	–	–	12	6
21	Universität Köln	–	–	–	–	–
22	Universität Leipzig	4	ja	–	–	8
23	Universität Lübeck	4	–	–	–	8
24	Universität Magdeburg	4	–	––	–	5
25	Universität Mainz	–	–	–	12	6
26	Universität Marburg	6	–	–	–	6
27	Universität München	6	4	33	6	6
28	Universität Münster	–	–	–	–	5
29	Universität Oldenburg	2	–	–	–	–
30	Universität Regensburg	–	–	–	–	–
31	Universität Rostock	5	2	60	10	5
32	Universität Saarlandes	5	3	40	10	6
33	Universität Tübingen	2	–	–	–	6
34	Universität Ulm	2	–	–	12	6
35	Universität Würzburg	6	4	33	12	6

The table shows the detailed conditions, which could be found online. Therefore, it is important to note that an update of the homepages is necessary.

**Figure 1:**
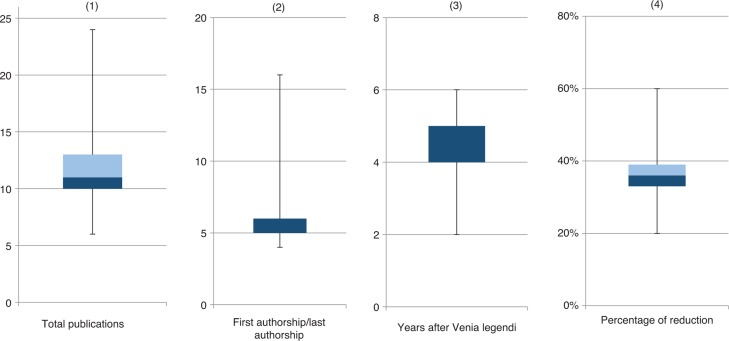
An average of 11 total publications is required after habilitation (median x̅=10, max=24, min=6, n=16) (1). The proportion of the first authorship/last authorship resulted in an average of 6 publications (x̅=6, max=16, min=4, n=26) (2). In most German universities, an average of 4 years is required to obtain a professorship (x̅=5 years, max=6 years, min=2 years) (3). At 10 universities, this period can be shortened by 38% on average (x̅=37%, max=60%, min=20%) (4). The conditions of shortening this time period are mainly linked to the listing of vacancies as an ordinary (clinical director) or outstanding researcher. The meaning of outstanding research is not further defined.

For earning a medical professorship, an average of 11 total publications is required after habilitation (median x̅=10, max=24, min=6, n=16; Figure 1, 1), whereas 6 publications with first or last authorship are required on average (x̅=6, max=16, min=4, n=26; Figure 1, 2). In most German universities, an average of 4 years after habilitation is required to obtain a professorship (x̅=5 years, max=6 years, min=2 years; Figure 1, 3). In 10 universities, this period can be reduced on average by 38% by applying for an extrainstitutional professorship (x̅=37%, max=60%, min=20%; Figure 1, 4). The conditions of shortening this period are mainly linked to the listing of vacancies as an clinical director or “outstanding researcher”. The meaning of “outstanding research” is not specified.

It is worth of note that some universities define their scientific achievements based on minimum achieved impact factors. For example, the Ludwig-Maximilians-University Munich has defined a point score by grading journals to qualitative criteria (IF>20: 4 points; top journal: 2 points; standard journal: 1 point). Hereby, the journals within the first 20% of the ranking list apply as “top journals” and the further 40% (between 20% and 60%) are declared as standard journal [19]. The scientific achievements required for the regular procedure require at least, on average, two IF points per year since habilitation. At the majority of universities, however, the impact factor or publishing in well-recognized journals plays no significant role.

Successful fundraising is another criterion gaining expected scientific achievements. Specifically, 10 of 35 universities state that third-party funds should be recruited as a prerequisite. The prerequisite of quality and grading of the doctoral thesis is necessary solely at RWTH Aachen University with the need of an excellent result. In summary, the condition of habilitation and professorship varies considerably between German universities.

## Discussion

Data show that the characteristics of universities to fulfill all criteria to obtain a professorship vary widely in Germany. Therefore, there are distinct advantages of becoming a full professor as mentioned above. Nevertheless, most of the researchers continue research because they see the value within the work. If one remains an apl professor, he still is in subordinate position in relation to the full professor (W2/W3) especially in Germany. Full professors (W2/W3) can delegate tasks and possible research topics and are more obligated to the management of the department, whereas the apl professorship is a no paid faculty position.

The occupation of a chair is a rare event in Germany. New professorships are scarce, and existing chairs are only announced after retirement of a professor. However, surveys in Germany still showed huge interests of students for an academic career [20]. Based on the different requirements within Germany as well as in an international comparison, the conclusion is that the conditions to obtain a professorship are very different. The time for obtaining the German “Privatdozent” equivalent to assistant professorship takes about 4 years after specialization and an additional 4 years for obtaining an apl professorship in the field of plastic surgery [1]. Besides outstanding research with excellent results measured by impact factor as well as the development of treatment options, further publications of great academic importance are necessary. However, a direct promotion for a full professorship is possible if one is appointed for a university chair position. Finally, a professor has proven to be able to obtain funding for research.

From the perspective of the responsibilities performed by an academic, they are expected to publish research, books, or book chapters, teach, edit journals, and apply for grants to raise funds while supervising students. Besides the academic tasks, the clinical care of patients and teaching take an important part.

The balance of patient care, teaching, and research should be an important health policy goal. Therefore, teaching of current research results and presentation of clinical patient cases in the context of student education are necessary. Modern therapies and approaches must therefore be conveyed in a high-quality manner. Didactic education should also be extended. Further education in competences in the field of pedagogy and in the field of teaching is essential and should be further invested in.

Especially, academic tasks generate grants and research-associated money. In addition, major grants are a fundamental financing base for universities. The ambition of gaining a professorship for whatever personal reason is necessary to secure the research continuation that affects the grant income. Keeping this idea and concept in mind, many students, interns, and postdocs begin to choose to work at a department. By having obtained a full professorship, it represents a scientific, intellectual, socially exposed position and, perhaps, a fundamental intellectual superiority.

To maintain the attractiveness of medical academic career, the tenure track program was established as promotion to young scientists, with the aim to make the career steps more transparent and more predictable. The tenure track is a system widely used in the U.S. education system for recruiting lifelong university staff. A professor is initially employed on a temporary base. He is academically independent but is subject to a continuous performance requirement and control to get a prospect of tenure. This career design is expressed as a tenure track getting a fixed-term contract (usually 6–7 years) as an assistant professor with clear targets for the contract period and a fixed career promise in the case of probation. Within the scope of this fixed-term contract, undergoing the program requires an increased expense before becoming an associate or full professor. The prerequisite for a tenure position at a U.S. university is, in particular, an extensive list of publications, the acquisition of third-party funds, positive evaluation by students, and the commitment in the faculty and university [21].

Based on the U.S. system, tenure track is established in Germany by introducing junior professorship since the Fifth Amendment to the Higher Education Framework Act of 2002. However, there is a negative attitude among German higher education authorities, as one erroneously assumes the program as a “regular promotion”. As a rule, junior professorship usually runs for a limited period of 3–6 years. A further demand is that career paths at German universities should become “internationally more comprehensible” and “more transparent” [15].

However, the establishment of junior professorship with tenure track was not completely implemented as analyses showed that there are not as many positions existing as required. Junior professorship has a bad reputation among other scientists. According to a recent survey, almost half of them complained about the poor predictability of their careers [22], [23]. Another comparison survey of 604 junior professors showed that different career paths among junior professorship are better than its reputation [13], [24]. Eighty-five percent of respondents claimed that they would once again decide for a junior professorship, whereas 90% value the freedom in research and 77% are satisfied with the work tasks and content [13]. Critics said that the career path also works just without a stay perspective. After all, the faculty might want to choose another focus after 6 years or does not want to continue the contract for cost reasons. Universities also fear to secure a career perspective at an early stage for scientists being not suitable for a professorship in the end.

A direct and not a research-connected purchase of professor titles is also possible. Particularly in Eastern Europe, providers of doctor and professor titles offer the possibility of gaining a title such as “Professor honoris causa” leading to a misdirected system, in which titles may be misused for private practice or even within company names without any correlation to scientific achievements [25]. All of this leads to a devaluation of the title of professor. One can hardly judge if an individual is a professor by profession or only by title, if the professorship is the result of merits gained at a university, or if the title is only given by announcement. However, recent surveys showed benefits in clinical research fellowship programs with an increase in clinical research contribution [26].

## Conclusion

The present study shows the diversity and clearly different prerequisites for reaching a professorship within Germany at different universities and in comparison to the United States. Academic research productivity is closely linked to international exchange programs and fellowships. Although national and international academic exchanges are increasing international standardization, quality assurance and comparability are still missing. To gain a comparability of academic paths, an international academic program and a standardized academic program therefore should be implemented.

## Supplementary Material

The article (iss-2019-0011) offers reviewer assessments as supplementary material.
