# Supplementary material for: How to become a medical professor – a comparative analysis of academic requirements in Germany and the United States
Source: Innov Surg Sci. 2019 Aug 22;4(3):108–15. doi: 10.1515/iss-2019-0011 (PMC6817728; doi:10.1515/iss-2019-0011)
Supplement: Supplementary file 1 [file iss-4-20190011-s001.pdf]

Original Article

Seyed Arash Alawi*, Rosalia Luketina, Nicco Krezdorn, Lukas Fabian Busch, Anne Limbourg, Ludwik Branski, Peter M. Vogt and Andreas Jokuszies

How to become a medical professor – a comparative analysis of academic requirements in Germany and the United States

https://doi.org/10.1515/iss-2019-0011
Received June 11, 2019; accepted August 6, 2019

***Corresponding author: Seyed Arash Alawi,** Department of Plastic, Aesthetic, Hand and Reconstructive Surgery, Hannover Medical School, Carl-Neuberg-Strasse 1, 30625 Hannover, Germany, Phone: +49 511 532-8864, E-mail: alawi.seyed-arash@mh-hannover.de

Reviewers' Comments to Original Submission

Reviewer 1: Markus K. Diener

Jun 29, 2019

**Reviewer Recommendation Term:**	**Revise with Major Modifications**
**Overall Reviewer Manuscript Rating:**	50
Is the subject area appropriate for you?	5 - High/Yes
Does the title clearly reflect the paper's content?	1 - Low/No
Does the abstract clearly reflect the paper's content?	3
Do the keywords clearly reflect the paper's content?	3
Does the introduction present the problem clearly?	4
Are the results/conclusions justified?	4
How comprehensive and up-to-date is the subject matter presented?	4
How adequate is the data presentation?	4
Are units and terminology used correctly?	4
Is the number of cases adequate?	N/A
Are the experimental methods/clinical studies adequate?	2
Is the length appropriate in relation to the content?	5 - High/Yes
Does the reader get new insights from the article?	3
Please rate the practical significance.	N/A
Please rate the accuracy of methods.	4
Please rate the statistical evaluation and quality control.	4
Please rate the appropriateness of the figures and tables.	4
Please rate the appropriateness of the references.	4
Please evaluate the writing style and use of language.	5 - High/Yes
Please judge the overall scientific quality of the manuscript.	3
Are you willing to review the revision of this manuscript?	Yes

Open Access. © 2019 Alawi S.A., et al., published by De Gruyter. This work is licensed under the Creative Commons Attribution 4.0 Public License.

Comments to Author:

Dear authors,
I read your manuscript with great interest, and I do agree, there is much confusion on requirements for apl professorships etc. in Germany. So I do think, and this is in line with your conclusions, that transparency and standardization would be nice, nationally and internationally. Even if I completely agree with your conclusion, I do not agree with several components of your manuscript, the way towards your conclusions. My thoughts point-by-point in the following
1. Title: your title suggest a general workup of academic medicine; it is somewhat confusing the, when the reader gets to know in the introduction section that you focus on plastic surgery only. Focussing on plastic surgery should be displayed in the title already.
2.Scientific question: even if this represents a narrative overview, a clear scientific question should be stated an answered in the following. The manuscript in its current version is lacking a central theme.
3. There is much confusion of examples from plastic surgery and general statements of academic requirements in the field of medicine. It is unclear which data support the general comments and conclusions or if data support conclusions derived from plastic surgery only.
4. Within this context the methodology section does not clearly state whether all fields of academic medicine were evaluated or only plastic surgery components were analyzed.
5. Represantiveness of plastic surgery for the whole field of academic medicine must be discussed more thoroughly. Plastic surgery is is a surgical field which carries plenty opportunities to leave academy at some point and to focus on private practice. This is not the case in multiple other fields of medicine.
6.Since the research question is not defined properly as mentioned above, the additional comparison with the US system brings further noise into the manuscript. I really do recommend to focus on a central theme, a single question to answer in this manuscript. Only then, clear conclusions can be derived from proper methodology.

Reviewer 2: anonymous

Jun 23, 2019

**Reviewer Recommendation Term:**	**Revise with Major Modifications**
**Overall Reviewer Manuscript Rating:**	N/A
Is the subject area appropriate for you?	3
Does the title clearly reflect the paper's content?	4
Does the abstract clearly reflect the paper's content?	4
Do the keywords clearly reflect the paper's content?	4
Does the introduction present the problem clearly?	4
Are the results/conclusions justified?	3
How comprehensive and up-to-date is the subject matter presented?	3
How adequate is the data presentation?	4
Are units and terminology used correctly?	4
Is the number of cases adequate?	N/A
Are the experimental methods/clinical studies adequate?	N/A
Is the length appropriate in relation to the content?	3
Does the reader get new insights from the article?	3
Please rate the practical significance.	3
Please rate the accuracy of methods.	N/A
Please rate the statistical evaluation and quality control.	N/A
Please rate the appropriateness of the figures and tables.	2
Please rate the appropriateness of the references.	4
Please evaluate the writing style and use of language.	4
Please judge the overall scientific quality of the manuscript.	3
Are you willing to review the revision of this manuscript?	Yes

Comments to Author:

In the current paper the authors analyzed the requirements for medical professors in Germany with. Due to an online research, the authors queried German medical faculty regulations of 35 German universities to obtain a medical professorship within Germany. They revealed that 11 publications (in average) are required after the "Venia legendi" to obtain a professorship with 6 publications as a first or last author. In addition the authors showed that it takes an average of 4 years after receiving the "Venia legendi" to apply for a professorship that can be shorten candidates for university chair positions. Based on these findings the authors underline that there is currently no national or even international standardization, quality assurance and comparability to receive a medical professorship.

In my opinion the authors discuss a very interesting issue that is well analyzed and discussed.
However, a professorship, especially a surgical professorship labels three major categories: patient care, research and student teaching. In fact, the authors did almost not mention or analyse student teaching as a requirement for a medical professorship. Therefore, an additional analysis in the paper for that highly important field is needed.
A minor comment is about the tables: the graphic quality should be optimized.

Authors' Response to Reviewer Comments

Jun 10, 2019

Dear reviewer,
thank you for your letter and constructive comments concerning our manuscript entitled "How to become a medical professor –Comparative analysis of academic requirements in Germany and the United States. ". We have studied your comments carefully and made major correction which we hope meet with your approval. We answer your questions or comments in details in the following texts and marked the changes in track-change modus. Detailed answer to review:

Reviewer 1 :
1. Title: your title suggest a general workup of academic medicine; it is somewhat confusing the, when the reader gets to know in the introduction section that you focus on plastic surgery only. Focussing on plastic surgery should be displayed in the title already.

Reply: Our manuscript and the analyzed data is not focused on plastic surgery. We analyzed the variable requirements for prospective medical professors in Germany with special focus on the tenure track concept and the U.S. system. A special focus is not set. However as an example plastic surgery is mentioned but is not subject of the investigation.
Page 1: Based on this we did not change the title "How to become a medical professor– A comparative analysis of academic requirements in Germany and the United States."
As an example academic development in plastic surery is mentioned. Page 3: "For example, among all members of German plastic surgery departments, about 14% of DGPRAEC (Deutsche Gesellschaft für Plastischen, Rekonstruktiven und Ästhetischen Chirurgen) German Society of Plastic, Reconstructive and Aesthetic Surgeons) complete their habilitation, and about 7% reach an associate professorship/full professorship [1]."

The manuscript is focued more generally on the academic system but describes also some characteristics in plastic surgery in the introduction, however the data is focused on the requirements for academic careers at medical faculties.
To clarify your valuable point we made following adjustment at the end oft he introduction:
"The conditions for the academic career with habilitation and obtaining professorship are equivalent for all medical subjects of a faculty.

As the requirements to become a medical professor vary in Germany we aimed to analyze variable requirements for prospective medical professors in Germany. The listed data apply to all medical disciplines of the respective faculties.

Based on an online research we queried German medical faculty regulations to obtain a medical apl-professorship within Germany. We analyzed the variable requirements for prospective medical professors in Germany."

2.Scientific question: even if this represents a narrative overview, a clear scientific question should be stated an answered in the following. The manuscript in its current version is lacking a central theme.

Reply: We have stated the objective of our work
thoroughly and chosen in. Starting in the abstract we write:
"BACKGROUND The acquisition of a medical professorship represents a significant step in a physician's academic career. The responsibility as well as the honor and the associated obligations are significant, however, the requirements to become a medical professor vary in Germany.
OBJECTIVE We analyzed the variable requirements for prospective medical professors in Germany with special focus on the tenure track concept and the U.S. system."
In the introduction, we made the goal-oriented scientific question: "As the requirements to become a medical professor vary in Germany we aimed to analyze variable requirements for prospective medical professors in Germany. Based on an online research we queried German medical faculty regulations to obtain a medical apl-professorship within Germany. We analyzed the variable requirements for prospective medical professors in Germany."
3. There is much confusion of examples from plastic surgery and general statements of academic requirements in the field of medicine. It is unclear which data support the general comments and conclusions or if data support conclusions derived from plastic surgery only.

Solely two parts mention the situation in plastic surgery. It is more a kind of example fort he topic of academic career.
Introduction:
Page 3 “For example, among all members of German plastic surgery departments, about 14% of DGPRAEC (Deutsche Gesellschaft für Plastischen, Rekonstruktiven und Ästhetischen Chirurgen) German Society of Plastic, Reconstructive and Aesthetic Surgeons) complete their habilitation, and about 7% reach an associate professorship/full professorship”

Page 4 “From the perspective of the field of plastic surgery worldwide, we have gone through exciting and innovative developments, especially in the last decades. Plastic surgery departments of high output in Germany have developed a high level of specialization with an academic background that is defined in an independent academic environment within the university [7-9]. This independency is the base for academic careers. Additionally, funding and financial resources seem to be more available at university hospitals. Current developments include the establishment of a registry of research funding in the German Society of Plastic, Reconstructive and Aesthetic Surgeons (DGPRÄC) [10]. Currently, significant discrepancies exist between dependent and independent plastic surgery university hospitals regarding material and human resources. Investigations showed that the scientific performances of university hospitals are significantly better [8]. Regarding career steps, most of the habilitated physicians from German universities leave university hospitals before gaining a professorship [1]. The underlying facts in quitting the research field varies and may range from losing interest in research to “burn out” considered as not being able to exhibit the expected results. In addition, reorientation and the desire to work in a private practice count as further reasons [11, 12]. At the same time, the absence of financial and structural incentives leads to a lack of willingness to choose the stony path of obtaining a professorship. Analysis of German plastic surgery departments show that the intention to proceed with academic career stagnates after the accomplishment of habilitation [4].“

Page 7 :
At the end of the manuscript we added following:
“The conditions for the academic career with habilitation and obtaining professorship are equivalent for all medical subjects of a faculty.

As the requirements to become a medical professor vary in Germany we aimed to analyze variable requirements for prospective medical professors in Germany. The listed data apply to all medical disciplines of the respective faculties.

Based on an online research we queried German medical faculty regulations to obtain a medical apl-professorship within Germany. We analyzed the variable requirements for prospective medical professors in Germany.”

4. Within this context the methodology section does not clearly state whether all fields of academic medicine were evaluated or only plastic surgery components were analyzed.

Thank you for this comment :
We have completed this point

Page 8, Material and Methods:
“We carried out a web-based analysis of available online information about conditions of obtaining an apl-professorship based on the regulations of German medical faculties independent of the medical discipline. In order to work out the differences we evaluated both, the faculty regulations and federal state law and analyzed all updates concerning the subject. “

5. Represantiveness of plastic surgery for the whole field of academic medicine must be discussed more thoroughly. Plastic surgery is is a surgical field which carries plenty opportunities to leave academy at some point and to focus on private practice. This is not the case in multiple other fields of medicine.

Introduction Page 6,7
“The conditions for the academic career with habilitation and obtaining professorship are equivalent for all medical subjects of a faculty.
As the requirements to become a medical professor vary in Germany we aimed to analyze variable requirements for prospective medical professors in Germany. The listed data apply to all medical disciplines of the respective faculties.
Based on an online research we queried German medical faculty regulations to obtain a medical apl-professorship within Germany. We analyzed the variable requirements for prospective medical professors in Germany.”
Likewise in the abstract is clearly stated that it concerns the investigation of the entire academic medicine, since all requirement of medical faculties are examined. There is no limitation for plastic surgery.

Abstract:
“BACKGROUND The acquisition of a medical professorship represents a significant step in a physician’s academic career. The responsibility as well as the honor and the associated obligations are significant, however, the requirements to become a medical professor vary in Germany.
OBJECTIVE We analyzed the variable requirements for prospective medical professors in Germany with special focus on the tenure track concept and the U.S. system.”

6.Since the research question is not defined properly as mentioned above, the additional comparison with the US system brings further noise into the manuscript. I really do recommend to focus on a central theme, a single question to answer in this manuscript. Only then, clear conclusions can be derived from proper methodology.

The presentation of the academic US system serves the purpose of presenting the consequences as well as the adopted equations. Incidentally, the US is one of the main target countries for academic careers and also academic concepts are established. As already described in the manuscript, the German system has tried to establish many conditions and change the academic s system.

“In addition, Junior-professorships are connected with a tenure option based on the US academic system. The tenure track program for the promotion of young scientists should help to make the career paths in the academic world more transparent and to attract more university teacher careers in medicine. If successful, these positions should lead to a regular professorship without being publicly advertised again [14]. This also eliminates the time-consuming appointment procedure for a full professorship. The junior professorship was launched in 2002 with the fifth amendment of the Higher Education Framework Act (HRG). The objective of this amendment, initiated by Edelgard Bulmahn, the former federal minister of research, with the aim of making the German science system more competitive, especially at an international level [15, 16]. Due to the continuing brain drain the best minds were hired away of the German research and innovation location by other countries [17]. “

Page 3-4:
“In comparison to the German university system, individual universities in the US higher education system have a maximum autonomy in an output-oriented and competitive environment [5, 6]. This reflects the ongoing political discourse of the Federal Republic of Germany regarding future strategic orientation of universities being forced by the American model which appears to “strengthen university autonomy” and “introduce a board as a steering committee” [2, 5]. “

Dear reviewer,
thank you for your letter and constructive comments concerning our manuscript entitled “How to become a medical professor –Comparative analysis of academic requirements in Germany and the United States. ”. We have studied your comments carefully and made major correction which we hope meet with your approval. We answer your questions or comments in details in the following texts and marked the changes in track-change modus. Detailed answer to review:

Reviewer #2: In the current paper the authors analyzed the requirements for medical professors in Germany with. Due to an online research, the authors queried German medical faculty regulations of 35 German universities to obtain a medical professorship within Germany. They revealed that 11 publications (in average) are required after the “Venia legendi” to obtain a professorship with 6 publications as a first or last author.
In addition the authors showed that it takes an average of 4 years after receiving the “Venia legendi” to apply for a professorship that can be shorten candidates for university chair positions. Based on these findings the authors underline that there is currently no national or even international standardization, quality assurance and comparability to receive a medical professorship.

In my opinion the authors discuss a very interesting issue that is well analyzed and discussed.
However, a professorship, especially a surgical professorship labels three major categories: patient care, research and student teaching. In fact, the authors did almost not mention or analyse student teaching as a requirement for a medical professorship. Therefore, an additional analysis in the paper for that highly important field is needed.

The very important part of teaching is addressed at the following points in the manuscript but has also been completed based on your valuable comment.

Introduction:
Page 4/5
“Junior professors perform the same tasks as regular professors, responsibilities include in tutoring and supervising students, running third-party funded projects, perform committee work and administrative tasks. However, teaching load is reduced compared to full university professors(W2/W3).”

Page 5
“In Germany, teaching is one of the integral constituents of a “Privatdozent” after habilitation whom is further authorized to supervise doctoral thesis. One can apply for the position of apl-professorship equivalent to the associate professor in the U.S. after continuation of research and teaching [18]. “

Page 5: Introduction: We added:
“University hospitals and hospitals with university association have, besides the clinical patient care and research, also the task of teaching.

Teaching is an important part of academic work but often takes place alongside patient care and research activity. High-quality and sustainable knowledge transfer with the aim of promoting creative thinking processes and problem-oriented learning should is the aim of modern teaching concepts at university hospitals. The imparting of the ability to critically evaluate and solve medical questions continues to be a challenge for modern teaching institutions. Teaching activities are already graded at various universities. However, these have no relevance to the attainment of habilitation or for the further career steps at most of the universities. Nevertheless, teaching is required to obtain the various academic degrees and assessed on the basis of hours completed."

Page 10 , Discussion
"The balance between patient care, teaching and research should be an important health policy goal. Therefore, teaching of current research results and presentation of clinical patient cases in the context of student education are necessary. Modern therapies and approaches must therefore be conveyed in a high-quality manner. The didactic education should also be extended. Further education in competences in the field of pedagogy and in the field of teaching is essential and should be further invested in."

A minor comment is about the tables: the graphic quality should be optimized.
Reply: Thank you, we addressed this point .

Reviewers' Comments to Revised Submission

Reviewer 1: Markus K. Diener

Jul 17, 2019

**Reviewer Recommendation Term:**	**Accept**
**Overall Reviewer Manuscript Rating:**	50
Is the subject area appropriate for you?	5 - High/Yes
Does the title clearly reflect the paper's content?	1 - Low/No
Does the title clearly reflect the paper's content?	4
Does the abstract clearly reflect the paper's content?	4
Do the keywords clearly reflect the paper's content?	4
Does the introduction present the problem clearly?	4
Are the results/conclusions justified?	4
How comprehensive and up-to-date is the subject matter presented?	4
How adequate is the data presentation?	4
Are units and terminology used correctly?	4
Is the number of cases adequate?	4
Are the experimental methods/clinical studies adequate?	N/A
Is the length appropriate in relation to the content?	5 - High/Yes
Does the reader get new insights from the article?	3
Please rate the practical significance.	3
Please rate the accuracy of methods.	N/A
Please rate the statistical evaluation and quality control.	N/A
Please rate the appropriateness of the figures and tables.	4
Please rate the appropriateness of the references.	4
Please evaluate the writing style and use of language.	4
Please judge the overall scientific quality of the manuscript.	3
Are you willing to review the revision of this manuscript?	Yes

Comments to Author:
Dear authors,
I read your manuscript with great interest, and I do agree, there is much confusion on requirements for apl professorships etc. in Germany. So I do think, and this is in line with your conclusions, that transparency and standardization would be nice, nationally and internationally. Even if I completely agree with your conclusion, I do not agree with several components of your manuscript, the way towards your conclusions. My thoughts point-by-point in the following
1. Title: your title suggest a general workup of academic medicine; it is somewhat confusing the, when the reader gets to know in the introduction section that you focus on plastic surgery only. Focussing on plastic surgery should be displayed in the title already.
2.Scientific question: even if this represents a narrative overview, a clear scientific question should be stated an answered in the following. The manuscript in its current version is lacking a central theme.

3. There is much confusion of examples from plastic surgery and general statements of academic requirements in the field of medicine. It is unclear which data support the general comments and conclusions or if data support conclusions derived from plastic surgery only.
4. Within this context the methodology section does not clearly state whether all fields of academic medicine were evaluated or only plastic surgery components were analyzed.
5. Represantiveness of plastic surgery for the whole field of academic medicine must be discussed more thoroughly. Plastic surgery is is a surgical field which carries plenty opportunities to leave academy at some point and to focus on private practice. This is not the case in multiple other fields of medicine.
6.Since the research question is not defined properly as mentioned above, the additional comparison with the US system brings further noise into the manuscript. I really do recommend to focus on a central theme, a single question to answer in this manuscript. Only then, clear conclusions can be derived from proper methodology.

Reviewer 2: anonymous

Jul 15, 2019

**Reviewer Recommendation Term:**	**Revise with Major Modifications**
**Overall Reviewer Manuscript Rating:**	N/A
Is the subject area appropriate for you?	3
Does the title clearly reflect the paper's content?	4
Does the title clearly reflect the paper's content?	4
Does the abstract clearly reflect the paper's content?	4
Do the keywords clearly reflect the paper's content?	3
Does the introduction present the problem clearly?	4
Are the results/conclusions justified?	3
How comprehensive and up-to-date is the subject matter presented?	3
How adequate is the data presentation?	4
Are units and terminology used correctly?	4
Is the number of cases adequate?	N/A
Are the experimental methods/clinical studies adequate?	N/A
Is the length appropriate in relation to the content?	4
Does the reader get new insights from the article?	3
Please rate the practical significance.	N/A
Please rate the accuracy of methods.	N/A
Please rate the statistical evaluation and quality control.	N/A
Please rate the appropriateness of the figures and tables.	3
Please rate the appropriateness of the references.	3
Please evaluate the writing style and use of language.	3
Please judge the overall scientific quality of the manuscript.	3
Are you willing to review the revision of this manuscript?	Yes

Comments to Author:
The authors have to be congratulated for their revised manuscript.

However, in the manuscript an analysis about the teaching requirements in order to gain a medical professorship was not performed. Moreover, the authors state the following in their paper: "Teaching activities are already graded at various universities.
However, these have no relevance to the attainment of habilitation or for the further career steps at most of the universities." In my opinion this statement is incorrect as teaching is nowadays an important requirement for a medical professorship in many universities. Therefore, the authors should perform the analysis and revise their manuscript.

Editor-in-Chief Comments to Final Decision

Since the authors responded to the suggestions of the reviewers during the first review process, the paper was accepted.
